# Disproportionality analysis to investigate fatal adverse events with immune checkpoint inhibitors: Proceed with extreme caution

**DOI:** 10.1073/pnas.2608648123

**Published:** 2026-09-08

**Authors:** Bruno Revol, Charles Khouri, Emanuel Raschi

**Affiliations:** ^a^ Pharmacovigilance Department, Centre Hospitalier Universitaire Grenoble Alpes, Université Grenoble Alpes, Grenoble 38043, France; ^b^ Inserm U1300, Hypoxie Physiopathologie Lab, Université Grenoble Alpes, Grenoble 38700, France; ^c^ Department of Medical and Surgical Sciences, Alma Mater Studiorum–University of Bologna, Bologna 40126, Italy

We read with interest the Research by Sun and colleagues (1), who investigated the association between immune checkpoint inhibitors (ICIs) and fatal adverse events (AEs) reported in multiple pharmacovigilance databases (FAERS, Vigibase, and CVARD). A number of issues relating to the rationale, design, and interpretation of this study require critical appraisal to facilitate understanding by readers who are not familiar with these kinds of data and their relevant limitations.

First, the novelty of findings is uncertain because the spectrum, timing, and clinical features of fatal AEs in patients treated with ICIs were documented in a 2018 systematic review, including Vigibase analysis (2). In addition, the potential predictive value of immune-related AEs (irAEs) with respect to overall survival is well known (3). Sun and colleagues aimed to develop a modified two-step process to identify higher-than-expected associations between treatment with an ICI and reported AEs in the first instance, and then between ICIs and fatal AEs, although the true added value of this design is unclear. Notably, the validation of FAERS signals through Vigibase and CVARD is counterintuitive considering the large overlap (expected and documented) among pharmacovigilance databases; signal validation requires integration in a broader framework with different data sources such as longitudinal healthcare databases (cohort studies, possibly using a target trial emulation approach).

Second, disproportionality statistics were calculated based on reported AEs, which are subject to various biases resulting from the variable and complex factors associated with the reporting to pharmacovigilance systems, such as the nature of the drug, the severity of AEs, temporal changes of prescribing habits, and/or effective drug exposure. Pharmacovigilance in oncology raises several additional challenges, including combination or sequential treatments, frailty, comorbidities, and concomitant medications, leading to confounding by indication and channeling bias. Therefore, the term “fatality” should be interpreted very cautiously, because many “high-mortality AEs” identified in the study by Sun et al. (e.g., respiratory failure, septic shock) could be considered as markers of terminal cancer progression rather than being related to treatment per se, contributing to the high mortality rate reported (~25%). Infectious diseases are notably outside the spectrum of irAEs and illustrate the aforementioned biases. In contrast, data from meta-analyses suggest that fatal irAEs can occur in 0.3 to 1.3% of patients treated with an ICI (2). We can therefore reasonably estimate that a large proportion of the fatal cases reported were not drug related or that the majority of nonfatal AEs were not reported.

Third, as Sun and colleagues pointed out, disproportionality analysis is a hypothesis-generating tool, and relevant findings cannot provide direct guidance for regulatory or clinical actions (e.g., formal contraindications) but require subsequent analytical investigations. The misinterpreted use of causal language (also called spin) may even be exaggerated in related citations, possibly resulting in unjustified alarm (4, 5).

As a final research consideration, we advocate for the rigorous and collective adoption of the READUS-PV guideline, which was specifically developed for reporting of disproportionality analysis, thus promoting the proactive design and publication of transparent high-value studies (6, 7), including in the era of immunotherapy (8).

## Data Availability

There are no data underlying this work.
